# Predicting Risk Areas of Classical Scrapie in China Based on Environmental Suitability

**DOI:** 10.1155/2023/2826256

**Published:** 2023-06-28

**Authors:** Hong Li, Haoju Pan, Le Xu, Suya Li, Shiyuan Li, Si Chen, Churiga Man, Li Du, Qiaoling Chen, Jianhua Xiao, Hongbin Wang, Fengyang Wang, Hongyan Gao

**Affiliations:** ^1^Hainan Key Laboratory of Tropical Animal Reproduction & Breeding and Epidemic Disease Research, Haikou Key Lab of Animal Genetic Engineering, School of Animal Science and Technology, Hainan University, Haikou 570228, China; ^2^Department of Veterinary Surgery, College of Veterinary Medicine, Northeast Agricultural University, Harbin, Heilongjiang Province, China

## Abstract

Classical scrapie is a transmissible spongiform encephalopathy that attacks the central nervous system of sheep and goats. Since its discovery in the 18th century, the disease has caused enormous economic losses and public health impacts in continental Europe. In the late 20th century, classical scrapie began to spread to places, such as Asia and the Americas, becoming a disease of global concern. In this study, based on prion occurrence records and high-resolution environmental layers, a risk assessment of classical scrapie in China was performed using a maximum entropy model. The model achieved an area under the curve value of 0.906 (95% confidence interval, 0.0883–0.0929). Sheep distribution density, road density, goat distribution density, minimum temperature of the coldest month, port density, and precipitation of the driest quarter were identified as important factors affecting the occurrence of classical scrapie. The risk map showed that potential high-risk areas in China were mainly located in Northeast China, North China, and Northwest China. This study can provide a valuable reference for the prevention of classical scrapie in China. According to the environmental variables and risk areas of classical scrapie, implementing monitoring and early warning measures in these areas is recommended to reduce the possibility of classical scrapie occurrence and transmission.

## 1. Introduction

Classical scrapie (subsequently referred to as scrapie) is a chronic infection of the central nervous system of sheep and goats. Diseased sheep show progressive neuropathy, such as ataxia, itching, and death [1, 2], with a case fatality rate of up to 100%. Prion, the causative agent of scrapie, is a protein virus that causes mutations in the central nervous system of sheep, resulting in neuronal death and spongy cavities in brain tissue and the central nervous system. Because no effective treatment is available, once an epidemic of scrapie occurs, the entire flock must be completely culled, which will have a huge impact on the economic benefits of the sheep breeding industry [3, 4]. Scrapie has plagued humans for a long time, and according to the relevant literature, scrapie frequently occurred in the European continent before the 18th century [5], with definite records of scrapie starting in Spain in 1732 [6]. By the middle to late 1900s, scrapie was the most severe and widespread disease, and this disease has been reported in the Americas, East Asia, Oceania, and Africa because of frequent trade in sheep [7–9]. For example, in 1983, a scrapie event occurred in imported sheep from the United Kingdom in Southwest China, with several sick sheep showing typical itching and brain tissue injury [10]. In the 21st century, the occurrence of scrapie in the United Kingdom, United States, and France has decreased since the implementation of the prion elimination program in the European Union [11–13]. However, in the era of globalization and increasing frequency of international trade, the risk of the global spread of scrapie is increasing, and there is a risk of cross-border transmission. In particular, China is among the top countries in terms of the amount of meat sheep worldwide, and Japan, a neighboring country of China, is experiencing an epidemic trend [14, 15], all of which provide the potential for the occurrence and spread of scrapie in China.

Usually, to effectively prevent the occurrence and spread of infectious diseases, early detection of the epidemic trajectory of infectious diseases and timely adoption of effective preventive and control measures are key. However, because prion infections cannot induce an immune response in organisms, they cannot be diagnosed using serological methods. The immunohistochemical, immunoblotting, and tissue imprinting methods developed for detecting scrapie are all postmortem diagnostic and confirmatory diagnostic methods, making it difficult to provide early warning detection and implement control measures. Therefore, the epidemiology techniques of spatiotemporal prediction or modeling have become effective alternatives. For example, Ancelet et al. [16] applied Bayesian shared spatial component models to analyze spatial variations in the risk of scrapie infection affecting sheep in Wales (United Kingdom); Arnold and Rajanayagam [17] used a back-calculation model to predict the prevalence and trends of classical scrapie in Great Britain, which showed a possible further increase in classical scrapie cases in Great Britain. Several studies have focused on scrapie epidemic trends in European regions [17–20]; however, very little information is available on the global occurrence of prion infection and whether the ecological environment in China has an epidemic potential for scrapie.

Ecological niche models use species distribution data and certain algorithms to predict the potential distribution of species at different spatial and temporal scales [21, 22]. The selection of candidate predictors benefits from established theoretical and clinical expertise in the model-building process. Particularly, note that transmissible spongiform encephalopathies (TSEs) can affect various animals, and some sick individuals can also spread the disease among populations. For example, sheep carrying scrapie pathogens are processed into meat-and-bone meals and fed to cattle, which can cause the occurrence and prevalence of bovine spongiform encephalopathy [23]; human consumption of food contaminated with bovine spongiform encephalopathy increases the risk of developing Creutzfeldt–Jakob disease [24]; bovine spongiform encephalopathy can also infect sheep and goats under natural conditions [25, 26], thereby exacerbating the occurrence and prevalence of scrapie [27]. Small ruminants, including sheep and goats, are natural reservoirs of scrapie. Recently, the number of live sheep worldwide has been concentrated in Asia [28], and the distribution of sheep and goats has become a nonnegligible risk factor for the spread of scrapie. Further exploration of the range of factors influencing the persistent presence of prions in the environment will facilitate the study of the mechanisms of the occurrence and transmission of scrapie [29]. In fact, most pathogenic microorganisms can be transmitted through environmental pathways, and bioclimatic variables are an important factor [30]. In addition to bioclimatic factors, the distribution of soil characteristics, including the percentage of clay respectively in topsoil, the percentage of organic carbon in topsoil, and the available water storage capacity of the soil, has been identified to play an important role in the transmission of prion diseases [31]. Although these factors have limited impact on the short-term dynamics of scrapie at arbitrary locations, they can reasonably constitute a scrapie risk model at large scales of time and space.

In this study, an ecological niche modeling method was first used to establish a scrapie risk model in China based on environmental suitability. The potential high-risk areas must be monitored with an emphasis in the future, which can help guide the implementation of epidemic disease prevention and control programs more effectively.

## 2. Materials and Methods

### 2.1. Collection and Processing of Prion Occurrence Data

In this study, 168 occurrence records of TSEs (including classical scrapie and bovine spongiform encephalopathy) were collected. This study recorded the aforementioned data as prion occurrence data. The main sources included the following four aspects:

1. Comprehensive and systematic literature was retrieved from Web of Science, Scopus, ScienceDirect, PubMed, and Chinese National Knowledge Infrastructure, among others.
2. The records were collected from the Ministry of Agriculture and Rural Affairs of the People's Republic of China (http://www.xmsyj.moa.gov.cn/yqfb/).
3. The records were collected from the OIE World Animal Health Information System (https://wahis.woah.org/#/home).
4. The records were collected from the European Commission (https://ec.europa.eu/info/index_en).

For prion occurrence records without coordinates but with detailed geographical locations, Google Maps was used to determine the geographical coordinates of these occurrence records. To minimize spatial autocorrelation and remove duplicate records, we used SDMtoolbox (version 2.2; http://sdmtoolbox.org/) to filter the occurrence records within 1 km, and finally, 128 occurrence data were determined (Table S1).

### 2.2. Collection and Processing of Environmental Variables

In the process of building the scrapie risk model, the natural and humanistic environmental variables considered are shown in Table 1. The natural environmental variables included 19 bioclimate variables (Bio 1–Bio 19) representing global climate conditions and were downloaded from WorldClim (version 2.1; https://worldclim.org/data/worldclim21.html#), and these variables have been widely used in spatial modeling of animal diseases [32–35]. In addition to bioclimate variables, the model also introduces soil characteristic data (i.e., available water storage capacity, percentage of sand, percentage of silt, percentage of clay, percentage of organic carbon, the acidity and alkalinity of the soil). Global soil characteristic data were obtained from Harmonized World Soil Database (version 1.2; https://www.fao.org/soils-portal/soil-survey/soil-maps-and-databases/harmonized-world-soil-database-v12/en/). The humanistic environmental variables included: (1) airport density, port density, railway density, and road density; the raw data were downloaded from Natural Earth (https://www.naturalearthdata.com/downloads/10m-cultural-vectors/), and then, the raster layers were generated by kernel density analysis in ArcGIS 10.2. (2) Host distribution variables, including sheep and goat distribution density, were obtained from FAO Livestock Systems (https://www.fao.org/livestock-systems/en/).

Because a multicollinear relationship between bioclimate variables will lead to overfitting of the model predictions [36], checking for multicollinearity between variables before modeling is necessary. First, a correlation test was performed between two variables based on the Spearman correlation coefficient (Spearman's *r*). When |*r*| ≥ 0.7, there is a strong correlation between variables [37–39]. Then, we included all bioclimate variables in the model for a prerun. According to the contribution percentage of bioclimate variables to the model and the Spearman coefficient, the variable with a small contribution percentage is removed among the two strongly correlated variables. Finally, 19 variables, including seven bioclimatic variables, six soil characteristics, and six humanistic environmental variables, were included in the scrapie model (Table 1).

Finally, we obtained the global and China standard maps from the public standard map service (http://bzdt.ch.mnr.gov.cn/) of the National Surveying and Mapping Geographic Information Bureau, and the figure number is GS(2016)1666 and GS(2019)1822, respectively. Then, all variables used in the prion model were clipped to a unified global spatial scope; their projection coordinate system was the World Geodetic System-1984 Coordinate System, and the resolution was 2.5 min of arc (approximately 5 km × 5 km). ArcGIS 10.2 was used to convert the variables to the ASCII format for MaxEnt modeling.

### 2.3. Establishing an Ecological Niche Model

The MaxEnt model is a powerful tool based on a machine learning algorithm that can predict the spatial distribution of species using the presence points and environmental variables [40]. This model has excellent prediction and early warning performance and is widely used to predict areas with high incidences of animal diseases [41, 42]. In this study, MaxEnt, version 3.2.0, was used to build the prediction model [43]. When running MaxEnt, 25% of the prion occurrence data were set as test samples (randomly selected by the program) to test the prediction accuracy of the model; the remaining 75% of the prion occurrence data were used as modeling samples for the training of the model. To reduce sampling bias, 10,000 background points were selected to be defined as “pseudo-absence” data. Model replicates were set 10 times, and the final output of the model was averaged from 10 replicates.

The jackknife test refers to the training gain generated for a model when each variable is applied individually for modeling [44]. Comparing the training gains of all variables helps determine which variable contributes the most to the model. The receiver operating characteristic (ROC) curve of the test data was used to evaluate the performance of the model, and the area under the curve (AUC) was the area enclosed by the ROC curve and the *X*-axis. The AUC values range from 0 to 1. AUC values ≤0.5 indicate that the performance of the final model is worse than that of random distribution. The larger the AUC value, the better the prediction effect of the model. The risk map of scrapie occurrence consists of a range of values from 0 to 1, representing the low to high risk of scrapie occurrence, respectively. The potential classical scrapie risk maps were visualized using ArcGIS 10.2.

## 3. Results

### 3.1. Variables Used in the Scrapie Model

**Figure 1:**
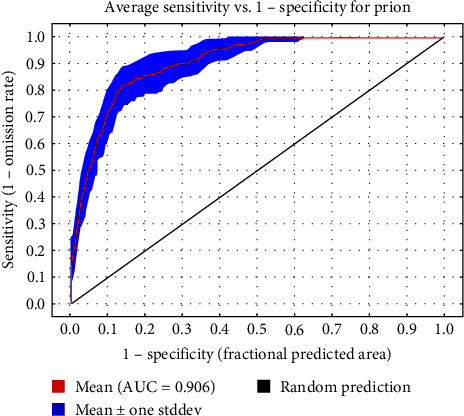
ROC curve of scrapie model.

The scrapie model showed good performance.  Figure 1 shows the ROC curve for the scrapie model, with an AUC of 0.906 and a standard deviation of 0.023.

**Figure 2:**
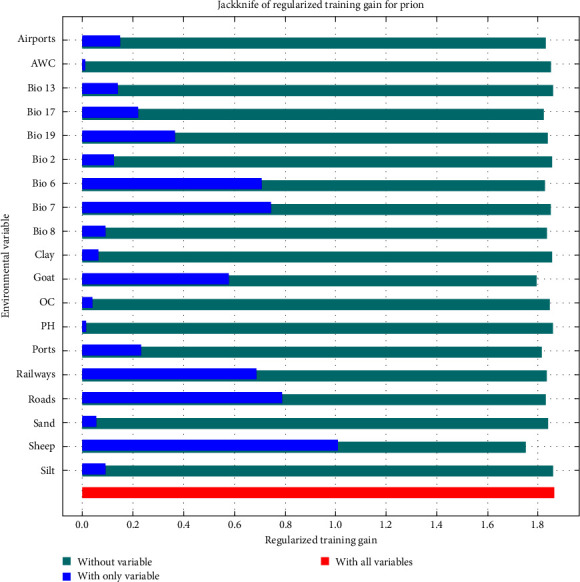
Jackknife test results of scrapie model.

Figure 2 shows the results of the jackknife test; the variables with the most gains have the most useful information [45]. For this model, sheep distribution density was the environmental variable with the highest gain when used in isolation, followed by road density, annual temperature range, minimum temperature of the coldest month, railway density, goat distribution density, precipitation of coldest quarter, port density, precipitation of the driest quarter, among others. Furthermore, sheep distribution density was the environmental variable whose gain decreased the most when it was omitted, followed by goat distribution density, port density, precipitation of driest quarter, minimum temperature of the coldest month, and road density. Collectively, the previous results revealed that the important factors affecting the occurrence of scrapie are sheep distribution density, road density, goat distribution density, minimum temperature of the coldest month, port density, and precipitation of the driest quarter.

**Figure 3:**
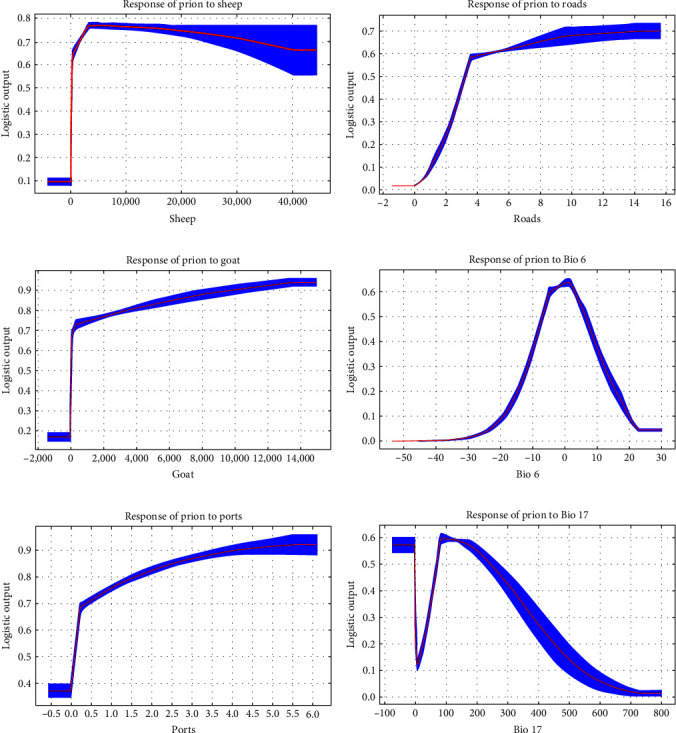
Response curves of important variables in scrapie model.

The response curve of important factors in the scrapie model is shown in Figure 3. The response curve of sheep distribution density showed that the probability of prion infection increased with an increase in sheep distribution density in the range of 0–3469 head/km^2^. According to the response curve of road density, the probability of prion infection increased with an increase in the road density index. The response curve of goat distribution density showed that goat distribution density was positively correlated with the probability of prion infection. The response curve of Bio 6 showed that the minimum temperature of the coldest month was between −10 and 10°C, which is relatively favorable for prion infection, and both lower and higher temperatures can decrease the probability of prion infection. The response curve of port density also showed that the port density index was positively correlated with the probability of prion infection. The response curve of Bio 17 showed that the precipitation of the driest quarter was from 0 to 87.8 mm, and the probability of prion infection increased sharply. However, when the precipitation exceeds 87.8 mm, with a gradual increase in precipitation, it is more unfavorable for prion infection.

### 3.2. Potential Risk Areas of Classical Scrapie in China

**Figure 4:**
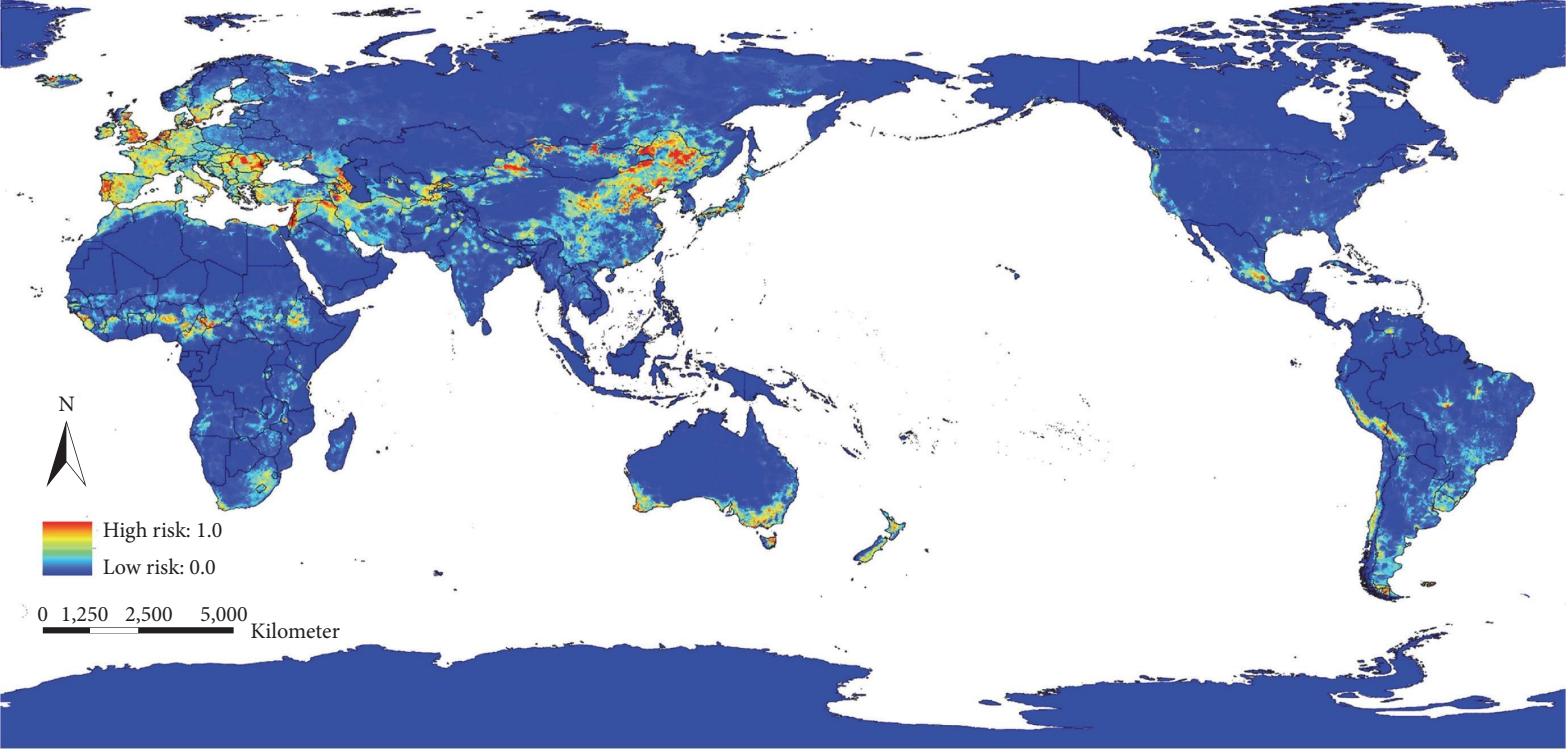
The global risk map of classical scrapie occurrence. The standard map was downloaded from the public standard map service of the National Surveying and Mapping Geographic Information Bureau, and the figure number is GS(2016)1666. The environmental suitabilities for global prion occurrence were marked on the map using ArcGIS, and the standard map has not been modified.

The predicted global risk areas of scrapie occurrence are shown in Figure 4. It can be seen from Figure 4 that the high-risk areas were mainly concentrated in the middle latitudes of the Eurasian continent, the Sudano–Sahelian zone, and coastal areas of southern Australia. Note that although there has been no scrapie outbreak in China, the predicted results showed that the risk of scrapie in China is widely distributed.

**Figure 5:**
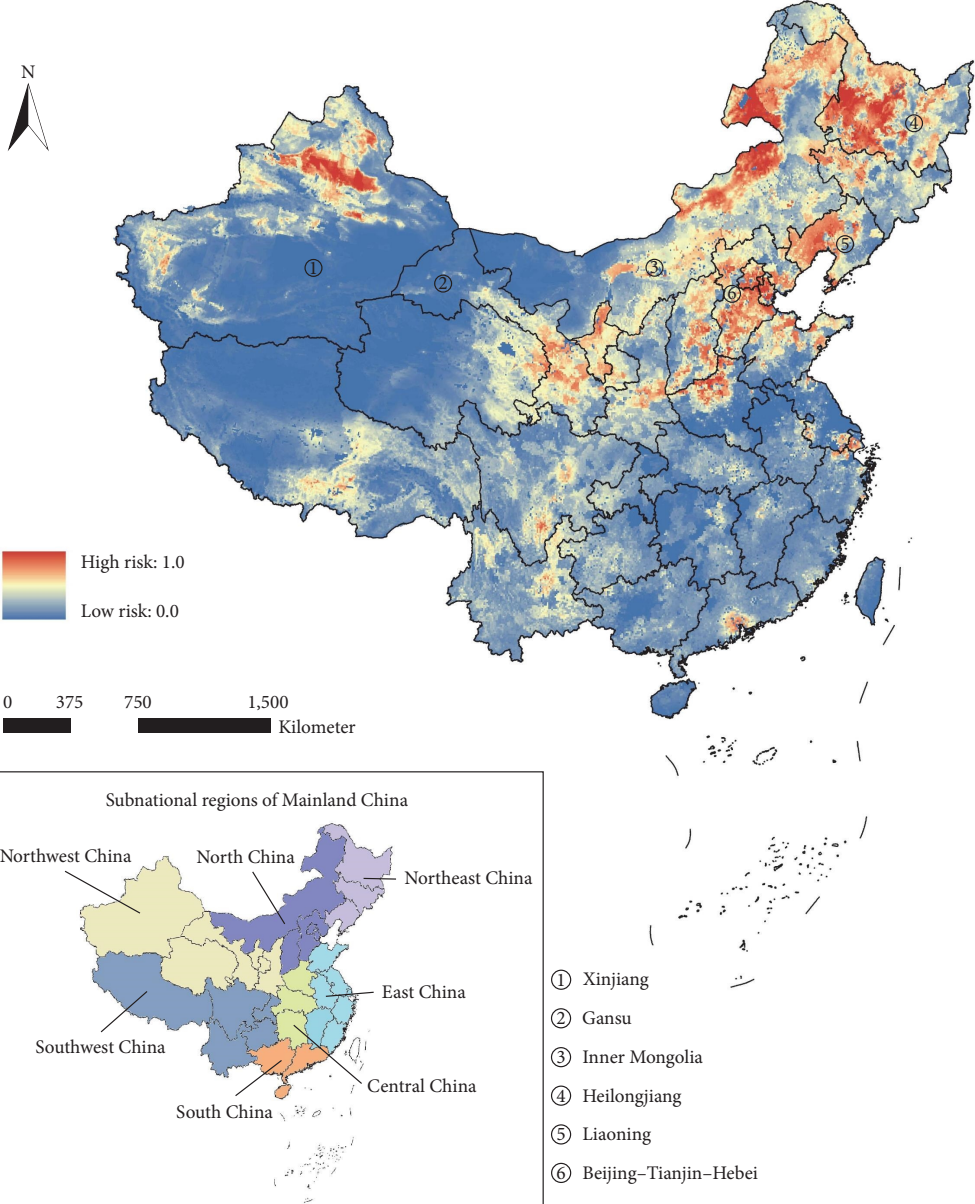
Potential risk areas of classical scrapie in China. The standard map was downloaded from the public standard map service of the National Surveying and Mapping Geographic Information Bureau, and the figure number is GS(2019)1822. The potential risk map was marked on the map using ArcGIS, and the standard map has not been modified.

The potential risk map of scrapie in China is shown in Figure 5. The risk map showed that potential medium-to-high-risk areas in China were widely distributed in various subnational regions of Northeast China, North China, and Northwest China. Among them, high-risk areas were concentrated in Heilongjiang, eastern Inner Mongolia, and northern Xinjiang. Additionally, western Liaoning and the Beijing–Tianjin–Hebei region were also in higher risk areas.

## 4. Discussion

In this study, a MaxEnt model was established using various environmental variables to identify risk areas of classical scrapie in China. According to the outputs of the model, sheep, and goat distribution density were important risk factors, and the scrapie epidemic may be closely related to flock management. Two large-scale surveys of scrapie in the United Kingdom showed that the risk of scrapie increased significantly with the denser distribution of sheep and the larger size of the flock, and the risk of scrapie was higher in grazing sheep than in captive sheep [46–48]. Our results also showed that the probability of scrapie occurrence increased with an increase in sheep and goat distribution density. China is a big sheep- and goat-breeding country; according to the statistics of the National Bureau of Statistics of China in 2021, the total number of sheep and goats in Northeast China, North China, and Northwest China was as high as 208 million. The total number of sheep and goats in Inner Mongolia, Xinjiang, and Gansu ranks among the top three in China. Performing risk-based surveillance in previous high-risk areas is recommended, and sampling sheep and goats in these areas is also necessary reasonably allocate the time, funds, and human resources required for investigation and provide an effective reference for local governments to prevent scrapie.

In the era of economic globalization, trade in live livestock and livestock products between countries is frequent. Previous incidents showed that some countries and regions, such as Australia, New Zealand, the Americas, and Oceania, have been threatened by the importation risk of scrapie [7, 8, 49]. Our response curve results also showed that transport and trade factors, such as roads and ports, are important factors affecting scrapie prevalence. However, according to a questionnaire survey, most Asian countries have not performed the import risk analyses of TSE [50], and even some countries imported by-products from cattle and ruminants from countries known to be infected with bovine spongiform encephalopathy, which brought greater challenges to the prevention and control of scrapie in Asia. The high-risk areas predicted in this study, such as the northern border region of China, border several countries such as Mongolia. The natural environment and social conditions in these areas are complex, and because of the lack of natural barriers, co-grazing often exists. A series of cross-border transmission events showed that the accidental invasion of a foreign animal disease would not only cause a huge blow and loss to the development of animal husbandry in China but also cause immeasurable damage to neighboring countries and even the entirety of Asia [51–53]. Therefore, implementing an extensive scrapie monitoring program along the northern border of China is particularly important.

This study also explored the effects of various climatic factors on scrapie occurrence, which helps deepen the understanding of the ecological needs of scrapie. Relevant studies have shown that temperature can induce prion to fold, and prion (PrP^C^) folds into a toxic scrapie form (PrP^SC^) [54]. In environmental transmission, the bioavailability of prion in soil may also be affected by temperature [29]. Furthermore, the climatic conditions with more precipitation are unsuitable for prion occurrence [55]. Our results showed that minimum temperature of coldest month and precipitation of coldest quarter were important factors affecting scrapie occurrence, which may be a reason why the risk of scrapie was higher in northern China than in southern China. Prion, similar to many other pathogens, readily spread as an interconnected system in the host and environment. According to the trophic cascade hypothesis, we speculate that under certain conditions, climate factors may also increase the risk of scrapie epidemic occurrence by affecting ecosystem productivity. These complexities highlight the importance of taking a “one health” approach when studying zoonotic diseases.

Although soil characteristic factors did not contribute much in the process of building the model, the distribution of prion, similar to that of *Burkholderia pseudomallei*, *Bacillus anthracis*, and other soil-dwelling bacteria, is influenced by the biochemical properties of soil [56]. In the early days, scientists have found that prion could remain infectious in soil for many years [57]. Various studies have shown that prion has a higher binding capacity with silt clay than with sand soil and is more easily adsorbed on soil with a larger clay fraction [55, 58]. Therefore, it is speculated that the increase in scrapie risk is related to the increase of in the clay fraction [59]. In contrast, the occurrence of scrapie is associated with the soil drainage capacity [48]. In detecting sand and clay rainwater filtrates in the environment, it was found that the amount of prion in clay filtrates was higher [48, 55]. More pieces of evidence also showed that prion can stably bind to soil minerals, and the ability of prion to spread through the mouth will be enhanced [60], and it is easy to survive in the rumen digestive tract of animals [61]. We speculate that the focus of scrapie may be hidden in a specific narrow range of conditions. Perhaps, soil characteristics affect the growth suitability of plants, or minerals affect the virulence of prion infection and thus determine the host immune homeostasis.

Classical scrapie is a serious threat to the healthy development of the sheep- and goat-breeding industry. Strengthening the attention to the prevention and control of disease, actively building an early warning and monitoring mechanism, prohibiting the import of ruminants and ruminant-derived feed from countries with relevant cases, and prohibiting illegal cross-border livestock trading, among others, are necessary. Future research can focus on the polymorphism of the prion protein gene and implement a breeding plan for scrapie resistance. Additionally, this study has limitations; although pseudo-absence data were generated to reduce the sampling bias, the accuracy of our results may be affected by any biases or missing data.

## Figures and Tables

**Table 1:** Description of the variables.

Variable	Description	Raw data sources	Included
Natural environmental			
Bio 1	Annual mean temperature	WorldClim	N
Bio 2	Mean diurnal range		Y
Bio 3	Isothermality		N
Bio 4	Temperature seasonality		N
Bio 5	Max temperature of the warmest month		N
Bio 6	Min temperature of the coldest month		Y
Bio 7	Temperature annual range		Y
Bio 8	Mean temperature of the wettest quarter		Y
Bio 9	Mean temperature of the driest quarter		N
Bio10	Mean temperature of the warmest quarter		N
Bio 11	Mean temperature of the coldest quarter		N
Bio 12	Annual precipitation		N
Bio 13	Precipitation of the wettest month		Y
Bio 14	Precipitation of the driest month		N
Bio 15	Precipitation seasonality		N
Bio 16	Precipitation of the wettest quarter		N
Bio 17	Precipitation of the driest quarter		Y
Bio 18	Precipitation of the warmest quarter		N
Bio 19	Precipitation of the coldest quarter		Y
AWC	Available water storage capacity of the soil	HWSD	Y
Sand	Percentage sand respectively in topsoil		Y
Silt	Percentage silt respectively in topsoil		Y
Clay	Percentage clay respectively in topsoil		Y
OC	Percentage of organic carbon in topsoil		Y
PH	The acidity and alkalinity of the soil		Y
Humanistic environmental			
Airports	Airport density	Natural Earth	Y
Ports	Port density		Y
Railways	Railway density		Y
Roads	Road density		Y
Sheep	Sheep distribution density	FAO	Y
Goat	Goat distribution density		Y

## Data Availability

The data used to support the findings of this study are included within the article and supplementary information.

## Supplementary Materials

Supplementary Materials Table S1: prion occurrence records.
