# Supplementary material for: Predicting Risk Areas of Classical Scrapie in China Based on Environmental Suitability
Source: Transbound Emerg Dis. 2023 Jun 28;2023:2826256. doi: 10.1155/2023/2826256 (PMC12016686; doi:10.1155/2023/2826256)
Supplement: Supplementary Materials — Table S1: prion occurrence records. [file 2826256.f1.doc]

**Table S1** **Prion occurrence records**

| **records** | **Address** | **Sources of data** |
| --- | --- | --- |
| prion | Macedonia,Greece | Web of Science, Scopus, ScienceDirect, PubMed |
| prion | Leicestershire,England | Web of Science, Scopus, ScienceDirect, PubMed |
| prion | Oslo,Norway | Web of Science, Scopus, ScienceDirect, PubMed |
| prion | Edinburgh,Scotland | Web of Science, Scopus, ScienceDirect, PubMed |
| prion | Aberfeldy,Scotland | Web of Science, Scopus, ScienceDirect, PubMed |
| prion | Hokkaido,Japan | Web of Science, Scopus, ScienceDirect, PubMed |
| prion | Picardy,France | Web of Science, Scopus, ScienceDirect, PubMed |
| prion | Cyprus | Web of Science, Scopus, ScienceDirect, PubMed |
| prion | Centre-Val de Loire,France | Web of Science, Scopus, ScienceDirect, PubMed |
| prion | Northwest Iceland | Agriculture and Rural Affairs of the People's Republic of China |
| prion | Guarda,Portugal | Agriculture and Rural Affairs of the People's Republic of China |
| prion | Sauðárkrókur,Iceland | Agriculture and Rural Affairs of the People's Republic of China |
| prion | Northeast Iceland | Agriculture and Rural Affairs of the People's Republic of China |
| prion | Lovec,Bulgaria | Chinese National Knowledge Infrastructure |
| prion | West Bank,Palestine | Chinese National Knowledge Infrastructure |
| prion | Braila,Romania | Chinese National Knowledge Infrastructure |
| prion | Hitias,TIMIS,Romania | Chinese National Knowledge Infrastructure |
| prion | Kjellerup,Viborg county | Chinese National Knowledge Infrastructure |
| prion | Sákeresztúr,Fejér county,Hungary | Chinese National Knowledge Infrastructure |
| prion | Divacˇa,Obalno-kraška,Slovenia | Chinese National Knowledge Infrastructure |
| prion | Cape Dolphin, East Falkland Islands | Chinese National Knowledge Infrastructure |
| prion | Nidavacha district, Slovenia | Chinese National Knowledge Infrastructure |
| prion | Kocevje,Slovenia | Chinese National Knowledge Infrastructure |
| prion | Oulu Province, Finland | Chinese National Knowledge Infrastructure |
| prion | Zurich,Switzerland | Chinese National Knowledge Infrastructure |
| prion | Curitiba,Parana,Brazil | Chinese National Knowledge Infrastructure |
| prion | Paraiba,Brazil | Chinese National Knowledge Infrastructure |
| prion | Cerdeira,Portugal | Chinese National Knowledge Infrastructure |
| prion | Haute-viena,France | Chinese National Knowledge Infrastructure |
| prion | Ain,France | Chinese National Knowledge Infrastructure |
| prion | Nakagawa,Hokkaido,Japan | Chinese National Knowledge Infrastructure |
| prion | Košický kraj,Slovakia | Chinese National Knowledge Infrastructure |
| prion | Ialomiţa County,Romania | Chinese National Knowledge Infrastructure |
| prion | Iasi County,Romania | Chinese National Knowledge Infrastructure |
| prion | Västra Finlands län,Finland | Chinese National Knowledge Infrastructure |
| prion | Wallonie,Belgium | Chinese National Knowledge Infrastructure |
| prion | Kafar Kama,Kineret,Israel | Chinese National Knowledge Infrastructure |
| prion | Bezet,Akko,Israel | Chinese National Knowledge Infrastructure |
| prion | Vatnshóll,Norðurland vestra(Húnaþing vestra),Iceland | OIE World Animal Health Information System |
| prion | Minni-Akrar,Norðurland vestra(Akrahreppur),Iceland | OIE World Animal Health Information System |
| prion | Syðri-Hofdalir,Norðurland vestra(Sveitarfélagið Skagafjörður),Iceland | OIE World Animal Health Information System |
| prion | Stóru-Akrar 1,Norðurland vestra(Akrahreppur),Iceland | OIE World Animal Health Information System |
| prion | Grænamýri,Norðurland vestra(Akrahreppur),Iceland | OIE World Animal Health Information System |
| prion | Meghni,Tataouine(Remada),Tunisia | OIE World Animal Health Information System |
| prion | Meda,Guarda(Mêda),Portugal | OIE World Animal Health Information System |
| prion | Almeida,Dsvr Do Centro,Portugal | OIE World Animal Health Information System |
| prion | Grófargil,Norðurland vestra(Norðurland vestra),Iceland | OIE World Animal Health Information System |
| prion | Alftagerdi,Skagafjarðar,Iceland | OIE World Animal Health Information System |
| prion | Vallanes,Skagafjarðar,Iceland | OIE World Animal Health Information System |
| prion | Urdir,Eyjafjarðar,Iceland | OIE World Animal Health Information System |
| prion | Stora-Grof ytri,Skagafjarðar,Iceland | OIE World Animal Health Information System |
| prion | RAHAT,Hadarom,Israel | OIE World Animal Health Information System |
| prion | Oita,Oita,Japan | OIE World Animal Health Information System |
| prion | Minami-ku,Fukuoka,Japan | OIE World Animal Health Information System |
| prion | Yatta,West Bank | OIE World Animal Health Information System |
| prion | Bani Naiem,West Bank,Palestinian Territory | OIE World Animal Health Information System |
| prion | Tqou',West Bank,Palestinian Territory | OIE World Animal Health Information System |
| prion | Kaleitsa,Lovec,Bulgaria | OIE World Animal Health Information System |
| prion | Idanha-a-aNova,Castelo Branco (2005-2009),Portugal | OIE World Animal Health Information System |
| prion | Negev desert near Kuseife,Hadarom,Israel | OIE World Animal Health Information System |
| prion | Dolejavas,Koper,Slovenia | OIE World Animal Health Information System |
| prion | Valbom, Pinhel, Guarda,Guarda (2005-2009),Portugal | OIE World Animal Health Information System |
| prion | Silistea,Braila,Romania | OIE World Animal Health Information System |
| prion | Tirlesti,Prahova,Romania | OIE World Animal Health Information System |
| prion | Ramnicelu,Braila,Romania | OIE World Animal Health Information System |
| prion | Orastioara de Sus,Hunedoara,Romania | OIE World Animal Health Information System |
| prion | Hitias,Timis,Romania | OIE World Animal Health Information System |
| prion | Kanagawa,Kanagawa,Japan | OIE World Animal Health Information System |
| prion | Maayya,Haifa,Israel | OIE World Animal Health Information System |
| prion | Hortobágy,Hajdu-Bihar,Hungary | OIE World Animal Health Information System |
| prion | Jászberény,Jasz-Nagykun-Szolnok,Hungary | OIE World Animal Health Information System |
| prion | Sákerestúr,Fejer,Hungary | OIE World Animal Health Information System |
| prion | Riezlern,Vorarberg,Austria | OIE World Animal Health Information System |
| prion | Colider,Mato Grosso,Brazil | OIE World Animal Health Information System |
| prion | Campo Azul,Minas Gerais,Brazi | OIE World Animal Health Information System |
| prion | Nova Canaä do Norte,Mato Grosso,Brazil | OIE World Animal Health Information System |
| prion | Porto Esperidiao,Mato Grosso,Brazil | OIE World Animal Health Information System |
| prion | Sertanópolis,Parana,Brazil | OIE World Animal Health Information System |
| prion | Ponoka County,Alberta,Canada | OIE World Animal Health Information System |
| prion | Edmonton,Alberta,Canada | OIE World Animal Health Information System |
| prion | Innisfail,Alberta,Canada | OIE World Animal Health Information System |
| prion | Barrhead county No.11,Alberta,Canada | OIE World Animal Health Information System |
| prion | Eraskobing,South ,Denmark | OIE World Animal Health Information System |
| prion | GIVRON,Ardennes,France | OIE World Animal Health Information System |
| prion | Plats,Rhone-Alpes,France | OIE World Animal Health Information System |
| prion | Kraiburg a. Inn,Bayern,Germany | OIE World Animal Health Information System |
| prion | Prädikow,Brandenburg,Germany | OIE World Animal Health Information System |
| prion | 14-031-00001,Brandenburg,Germany | OIE World Animal Health Information System |
| prion | Fethard,Tipporary,Ireland | OIE World Animal Health Information System |
| prion | Loughrea,Galway,Ireland | OIE World Animal Health Information System |
| prion | Tully. Corcreaghy, Dundalk, County Louth,Louth,Ireland | OIE World Animal Health Information System |
| prion | Lippenhuizen,Friesland,Netherlands | OIE World Animal Health Information System |
| prion | Odiliapeel,Noord-Brabant,Netherlands | OIE World Animal Health Information System |
| prion | Grijpskerko,Zeeland,Netherlands | OIE World Animal Health Information System |
| prion | Verran, Tua,Nord-Trondelag,Norway | OIE World Animal Health Information System |
| prion | Mirsk,Dolnoslaskie,Poland | OIE World Animal Health Information System |
| prion | DUME,Dsvr Do Norte,Portuga | OIE World Animal Health Information System |
| prion | Roci,Covasna,Romania | OIE World Animal Health Information System |
| prion | Deusu,Cluj,Romania | OIE World Animal Health Information System |
| prion | Adlesici,Novo Mesto,Slovenia | OIE World Animal Health Information System |
| prion | Villar de Plasencia,Extremadura,Spain | OIE World Animal Health Information System |
| prion | Jerez de los caballeros,Extremadura,Spain | OIE World Animal Health Information System |
| prion | Viniogra do Arriba,La Rioja,Spain | OIE World Animal Health Information System |
| prion | A Coruña,Galicia,Spain | OIE World Animal Health Information System |
| prion | EI Piero,Castilla Y León,Spain | OIE World Animal Health Information System |
| prion | EL SAHUGO,Castilla Y Leon,Spain | OIE World Animal Health Information System |
| prion | Camargo,Cantabria,Spain | OIE World Animal Health Information System |
| prion | Horcajo Medianero,Castilla Y Loon,Spain | OIE World Animal Health Information System |
| prion | Schwyz,Einsiedeln,Switzerland | OIE World Animal Health Information System |
| prion | Lauterbrunnen,Bern,Switzerland | OIE World Animal Health Information System |
| prion | Sankt Gallen,Sankt Gallen,Switzerland | OIE World Animal Health Information System |
| prion | SOMERSET, England,ENGLAND,United Kingdom | OIE World Animal Health Information System |
| prion | Auchindoir and Kearn,Scotland,United Kingdom | OIE World Animal Health Information System |
| prion | Marion County,Florida,United States of America | OIE World Animal Health Information System |
| prion | Perry County,Alabama,United States of America | OIE World Animal Health Information System |
| prion | Tulare, County,California,United States of America | OIE World Animal Health Information System |
| prion | Texas,Texas,United States of America | OIE World Animal Health Information System |
| prion | France/81190 Montauriol Tarn | European Commission |
| prion | France/43800 Chamalieres surLoire(Haute-Loire) | European Commission |
| prion | France/23700 Dontreix(Creuse) | European Commission |
| prion | France/11230 Corbieres(Aude) | European Commission |
| prion | M5,United Kingdom | European Commission |
| prion | France/76 260ETALONDESSeine-Maritime | European Commission |
| prion | France/33540SAUVETERRE DEGUYENNEGironde | European Commission |
| prion | Munster,Ireland | European Commission |
| prion | Northwest Territories,Canada | European Commission |
| prion | Kansas,United States of America | European Commission |
| prion | Castilla-La Mancha,Spain | European Commission |
